# The Key Role of Plant Hormone Signaling Transduction and Flavonoid Biosynthesis Pathways in the Response of Chinese Pine (*Pinus tabuliformis*) to Feeding Stimulation by Pine Caterpillar (*Dendrolimus tabulaeformis*)

**DOI:** 10.3390/ijms25126354

**Published:** 2024-06-08

**Authors:** Yanan Zhao, Tianhua Sun, Jie Liu, Ruibo Zhang, Yongjie Yu, Guona Zhou, Junxia Liu, Baojia Gao

**Affiliations:** 1 College of Forestry, Hebei Agricultural University, Baoding 071000, China; ya_nanzhao@126.com (Y.Z.); tianhua0001@163.com (T.S.); 18631394322@163.com (R.Z.); 18233807061@163.com (Y.Y.); zhouguona1976@163.com (G.Z.); bdljx1997@126.com (J.L.); 2 College of Agronomy, Hebei Agricultural University, Baoding 071000, China; liu15733220385@163.com

**Keywords:** feeding stimulation, transcriptome, metabolome, Chinese pine, flavonoid biosynthesis, plant hormone

## Abstract

In nature, plants have developed a series of resistance mechanisms to face various external stresses. As understanding of the molecular mechanisms underlying plant resistance continues to deepen, exploring endogenous resistance in plants has become a hot topic in this field. Despite the multitude of studies on plant-induced resistance, how plants respond to stress under natural conditions remains relatively unclear. To address this gap, we investigated Chinese pine (*Pinus tabuliformis*) using pine caterpillar (*Dendrolimus tabulaeformis*) under natural conditions. Healthy Chinese pine trees, approximately 10 years old, were selected for studying induced resistance in Huangtuliangzi Forestry, Pingquan City, Chengde City, Hebei Province, China. Pine needles were collected at 2 h and 8 h after feeding stimulation (FS) via 10 pine caterpillars and leaf clipping control (LCC), to simulate mechanical damage caused by insect chewing for the quantification of plant hormones and transcriptome and metabolome assays. The results show that the different modes of treatments significantly influence the contents of JA and SA in time following treatment. Three types of differentially accumulated metabolites (DAMs) were found to be involved in the initial response, namely phenolic acids, lipids, and flavonoids. Weighted gene co-expression network analysis indicated that 722 differentially expressed genes (DEGs) are positively related to feeding stimulation and the specific enriched pathways are plant hormone signal transduction and flavonoid biosynthesis, among others. Two TIFY transcription factors (*PtTIFY54* and *PtTIFY22*) and a MYB transcription factor (*PtMYB26*) were found to be involved in the interaction between plant hormones, mainly in the context of JA signal transduction and flavonoid biosynthesis. The results of this study provide an insight into how JA activates, serving as a reference for understanding the molecular mechanisms of resistance formation in conifers responding to mandibulate insects.

## 1. Introduction

When plants face biotic or abiotic stress, a series of physiological and biochemical changes are initiated in forming resistance via various mechanisms [1,2,3,4,5]. Due to the introduction of ecological regulatory concepts and advancements in omics technologies, the application of inherent plant resistance for regulating pest development has become a hot topic in as well as the ultimate goal of plant resistance research.

**Figure 1:**
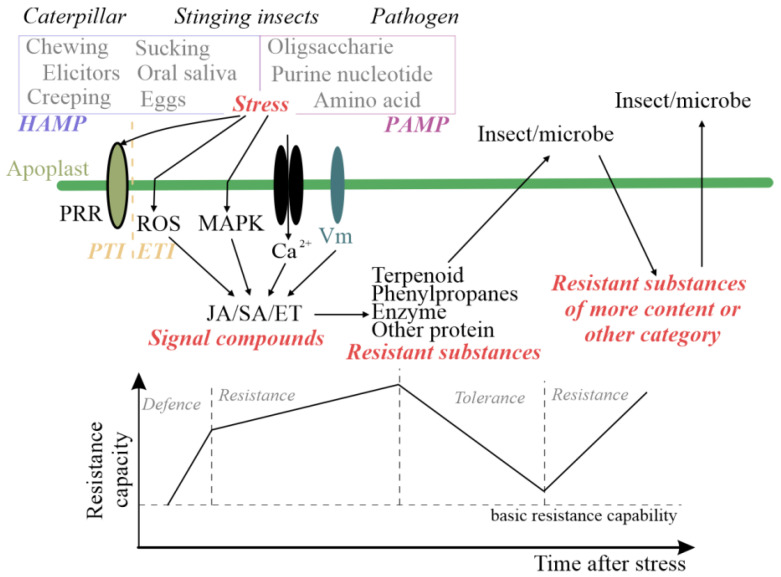
Schematic representation of plant-induced resistance [10,12,13]. Different colors are only used for distinction, with no other practical significance.

In plants, the induced defense response is activated only in the presence of stress and is more specific compared to constitutive defense responses; as such, it is considered the most advantageous approach for ecologically regulating of stress [6]. In accordance with the process of induced resistance, research has initially been focused on plant perception of herbivorous insects and pathogens. In early stages, insects can induce plants to initiate molecular patterns similar to pathogen-associated molecular patterns (PAMPs) when their mouthparts puncture or damage the plant during the feeding process. Apart from piercing–sucking and chewing, other responses during insect feeding trigger defense mechanisms that differ from those triggered against pathogens. These unique defense responses are known as herbivore-associated molecular patterns (HAMPs) [7]. As shown in Figure 1, the plant immune system activates pathogen-associated molecular patterns (PAMPs) or herbivore-associated molecular patterns (HAMPs), which trigger pattern-triggered immunity (PTI) through pattern-recognition receptors (PRRs) located on cell surfaces [8]. Intracellular nucleotide-binding domain leucine-rich repeat containing receptors (NLRs) can directly or indirectly recognize effector molecules and initiate effector-triggered immunity (ETI) signal transduction by activating effector-triggered immune responses [9]. In recent years, researchers have focused on optimizing and improving the “zig-zag model” [10], as shown in Figure 1, conceding that plant resistance cannot be adequately summarized using universal linear models [11]. The hormone signaling network connects perception and early signal transduction with extensive transcriptional reprograming and defense induction. Jasmonic acid (JA), salicylic acid (SA), and ethylene are among the most commonly found plant hormones [12].

After recognizing insect elicitors, plants can regulate the core components of downstream defense metabolites, such as glucosinolates, benzoxazinoids, cyanogens, alkaloids, phenolics, and proteases, in damaged leaves and whole plant tissues [14,15]. Furthermore, flavonoid compounds, alkaloids, defense enzymes, and resistance proteins are subsequently induced and play a significant role in antioxidant and insect resistance responses [16]. 

There has also been progress in research on the resistance of pine trees [17]. However, most studies on insects inducing resistance in trees have focused on indoor environments in an attempt to control other environmental factors [18]. There is a lack of comprehensive research on the expression of chewing insect-induced resistance under natural conditions, and the connection between plant hormone signaling and resistance substances remains relatively unclear. Whereas previous studies have examined the resistance of coniferous trees to insects primarily using spruce (*Picea*) and the white pine weevil (*Pissodes strobi*) as a model system [19,20], various pathogens and pests may employ distinct attack methods and mechanisms when targeting coniferous trees. With the genome sequencing of Chinese pine (*Pinus tabuliformis*) completed [21], many gene families involved in responding to biotic and abiotic stress have been identified [22]. Furthermore, there is a large number of introns, making Chinese pine a potential model plant for conifer breeding [23]. However, during the growth process of Chinese pine, there is an occurrence of Chinese pine caterpillar (*Dendrolimus tabulaeformis*), which severely inhibits the growth and development of Chinese pine forests. As a chewing insect with a specific habit of feeding on Chinese pine, researchers have extensively studied the feeding relationship between Chinese pine and the Chinese pine caterpillar, and the findings have been applied in various contexts. 

However, previous experimental studies have mostly been conducted indoors. It remains unknown whether insect foraging under natural conditions can induce defense responses in plants and what the patterns of these responses are. Which specific substances are induced in the defense response of conifer trees, and what is the upstream and downstream relationship between these substances? How do the metabolic pathways of resistance substances interact with other pathways? In this study, we attempt to answer these questions through experiments conducted under natural conditions, with the aim of providing a research foundation for identifying and understanding endogenous resistance substances and the mechanisms for their formation in conifer trees.

## 2. Results

### 2.1. Differences in Treatment Modes and Time after Treatment Impact on Plant Hormones

To clarify the extent to which treatment modes and time after treatment affect the formation of resistance, the JA and SA contents of pine needles in 0 h, FS 2 h, FS 8 h, LCC 2 h, and LCC 8 h groups were determined for gray relation analysis (GRA). The results demonstrate the main significant effects of treatment modes on defense (Table 1).

### 2.2. Analysis of FS-Induced Metabolites

**Figure 2:**
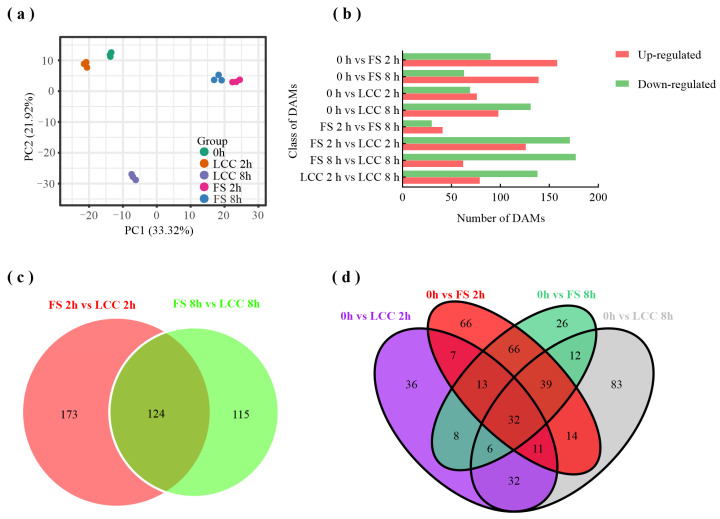
Basic information of DAMs. (**a**) PCA analysis results of metabolome between different samples. (**b**) Expression levels of DAMs. (**c**,**d**) Venn diagram of DAMs with different comparison groups.

To understand the overall metabolic differences between different treatments and the variability within each group, principal component analysis (PCA) was conducted on the metabolites. The results in Figure 2a show that PC 1 and PC 2 account for 33.32% and 21.92%, respectively, of the total variance. A distinct separation is observed between feeding stimulation and leaf clipping control, as well as a more significant distinction between LCC 2 h and LCC 8 h and between FS 2 h and FS 8 h. This result also suggests that, in addition to mechanical damage, there are specific elicitors that induce plant resistance during the feeding process. 

Using untreated pine needles (0 h) as the control, we screened for differentially accumulated metabolites (DAMs) in pine needles after different treatment modes and times after treatment. As shown in Figure 2b, the expression levels of some substances that were upregulated at 2 h tended to stabilize with the extension of feeding time. Moreover, feeding stimulation induced more metabolites than the leaf clipping control (FS 2 h vs. LCC 2 h and FS 8 h vs. LCC 8 h). Selecting DAMs with shared expression patterns among different comparison groups, we identified 124 substances showing differential expression in the 2 h and 8 h treatment groups (Figure 2c). Compared to 0 h, we found 32 DAMs that were co-identified in both FS and LCC treatments. Additionally, we observed 32 metabolites induced solely by caterpillars and another 32 metabolites induced solely by mechanical damage. Figure 2d presents the accumulation of a total of 66 DAMs specifically after feeding stimulation, with phenolic acids, lipids, and flavonoids being the top three substances. The expression trends of these DAMs are consistent between FS 2 h and FS 8 h.

**Figure 3:**
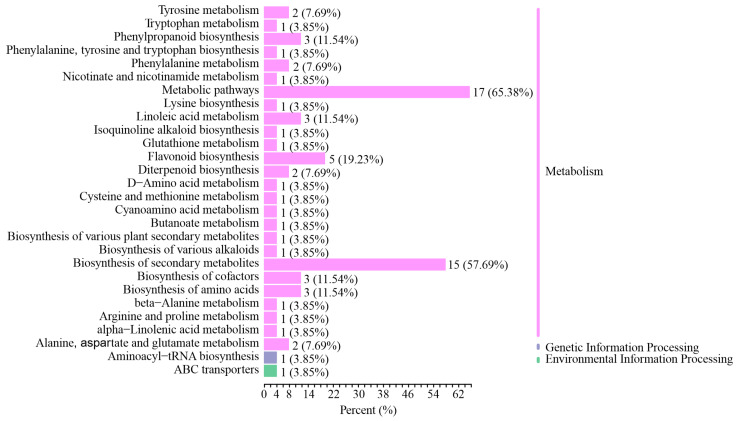
KEGG classification of DAMs induced by feeding stimulation.

The 66 DAMs specifically induced by feeding were further analyzed to confirm the identity of substance classes involved in resistance formation along with conducting KEGG annotation and clustering. The results (Figure 3) reveal significant enrichment in flavonoid biosynthesis, phenylpropanoid biosynthesis, linoleic acid metabolism, biosynthesis of cofactors, and the biosynthesis of amino acids in addition to metabolic pathways and the biosynthesis of secondary metabolites.

In summary, the results show that, in the early stages of feeding, there is an induced biosynthesis of substantial amounts of phenolic acids and other substances that are involved in the synthesis of flavonoids and phenylpropanoids. However, as the feeding continues, the production of these substances tends to stabilize.

### 2.3. Analysis of FS-Induced Genes

**Figure 4:**
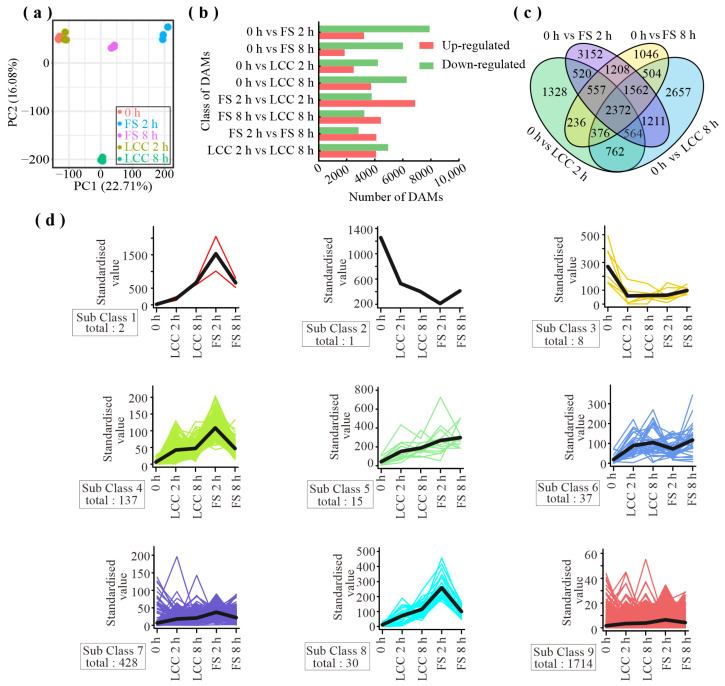
Overview of RNA-seq analysis. (**a**) Principle component analysis (PCA) of transcriptomes with different treatment modes and different times after treatment. Different colors represent different groups. (**b**) Number of upregulated DEGs and downregulated DEGs in the four comparison groups. Red indicates upregulated and green indicates downregulated. (**c**) Venn diagram of DEG distribution in the four comparison groups. Different colors represent different groups. (**d**) K-means analysis of co-expressed DEGs in the four comparison groups. Different colors represent different sub classes.

To explore which types of genes are regulated in pine needles after feeding stimulation, the same material was used to conduct RNA-seq analysis. A total of 108.62 GB clean data were collected. Following a comparison with the reference genome [21], differentially expressed genes (DEGs) were identified based on log2|Fold Change| ≥ 1 and FDR < 0.05. PCA showed the high reproducibility of biological repetition (Figure 4a). There were more genes downregulated than upregulated in FS 2 h vs. FS 8 h, but it was the opposite in LCC 2 h vs. LCC 8 h, which indicates that caterpillars can induce gene upregulation within shorter periods (0 h vs. FS 2 h), but that this increase does not persist with increasing time after treatment (Figure 4b). FS induced the differential expression of 4318 genes, of which 762 genes are co-expressed in 0 h vs. LCC 2 h and 0 h vs. LCC 8 h. LCC induced the differential expression of 4747 genes, of which 1208 genes are co-expressed in 0 h vs. FS 2 h and 0 h vs. LCC 2 h (Figure 4c). Then, K-means analysis resulted in clustering of the 2372 genes co-expressed in the four comparison groups into nine groups (Figure 4d). Only one gene (*Pt7G56800*) was downregulated in the LCC groups over time, but its expression in FS 8 h was higher than in FS 2 h.

**Figure 5:**
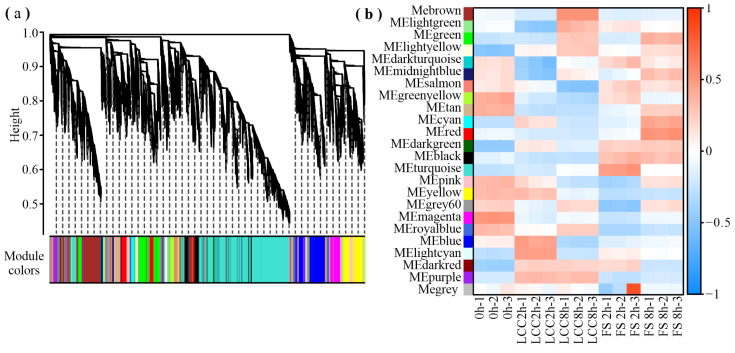
Cluster dendrogram (**a**) and module sample relationship (**b**) of the different modules. Different colors represent different modules.

As the hierarchical clustering of differentially expressed genes showed that the three replicates of each treatment clustered together, weighted gene co-expression network analysis (WGCNA) was conducted to precisely identify the genes related to feeding stimulation. Following clustering and significance calculations, 33,726 genes clustered into 23 modules: turquoise (9984), blue (2887), brown (2866), yellow (2288), green (2274), red (1716), black (1631), pink (1408), magenta (1214), purple (1023), and others (Figure 5a). The correlation coefficient determined between the module and sample, presented in black and purple, indicate positive and negative relationships to feeding stimulation (Figure 5b).

**Figure 6:**
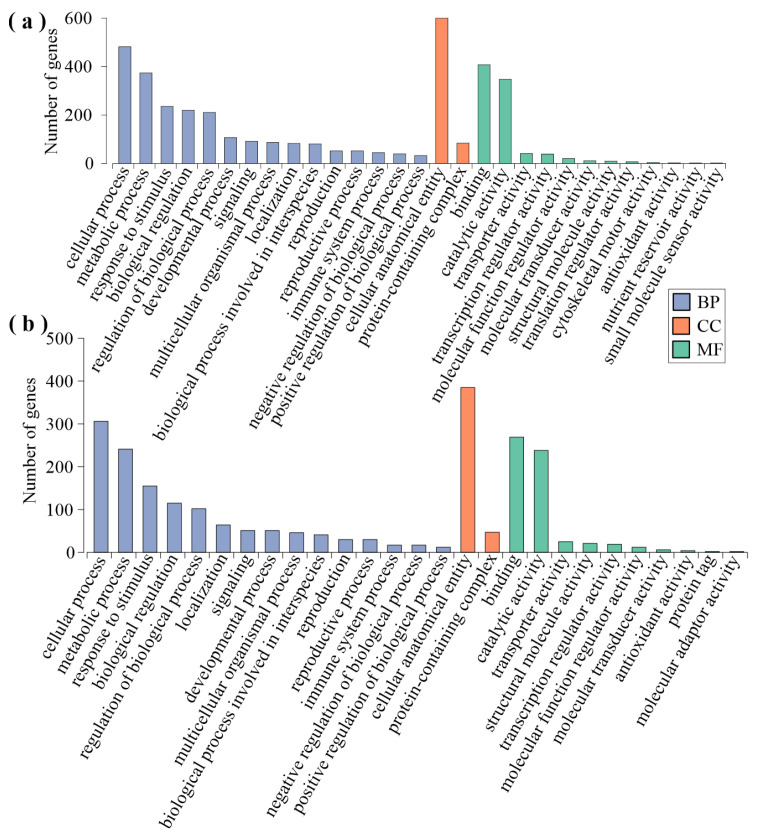
GO enrichment of DEGs in the black module (**a**) and purple module (**b**). Note: BP, biological process; CC, cellular component; MF, molecular function.

GO enrichment analysis was conducted on 722 genes with clear annotation information in the black module (Figure 6a). In the biological process (BP) category, the most enriched GO terms are cellular processes (482, 86.07%), followed by response to stimulus (237, 42.32%), macromolecular biosynthetic processes (123, 21.96%), and signaling (92, 16.43%). In the cellular component (CC) category, the most enriched group is intracellular parts (449, 73.73%), followed by membrane-enclosed lumen (51, 8.37%) and vacuolar membrane (23, 3.78%). In the molecular function (MF) category, the most enriched group is ion binding (269, 45.13%), followed by adenyl ribonucleotide binding (130, 21.81%) and hydrolase activity acting on acid anhydrides in phosphorus-containing anhydrides (41, 6.88%). GO enrichment analysis was conducted on 463 genes with clear annotation information in the purple module (Figure 6b). In the biological process (BP) category, the most enriched groups are response to stimuli (159, 43.32%), followed by cellular biosynthetic processes (112, 30.52%), cellular macromolecule biosynthetic processes (70, 19.07%), RNA metabolic processes (65, 17.71%), and signaling (52, 14.17%). In the cellular component (CC) category, the most enriched group is intracellular parts (268, 69.61%), followed by plasma membrane (96, 24.94%) and endoplasmic reticulum (24, 6.23%). Unlike in the black module, membrane-enclosed lumen and vacuolar membrane are not enriched in the purple module. In the molecular function (MF) category, the most enriched groups are transferase activity (102, 25.89%) and purine ribonucleotide binding (96, 24.37%). These two categories are also not enriched in the black module.

**Figure 7:**
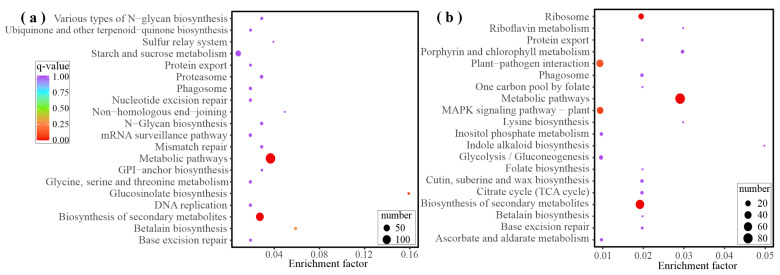
KEGG pathway enrichment of DEGs in the black (**a**) and purple (**b**) modules. The size of the point means number of DEGs enriched in the specific pathway and the color of the point means q-value of the pathway.

Due to the high number of DEGs in the black and purple modules in the comparison groups of feeding stimulation and leaf clipping control, KEGG enrichment analysis was conducted on the genes of these two modules to determine the metabolic differences caused by the treatments. The results, shown in Figure 7, indicate the top five enriched metabolic pathways for both modules. In the black module, the following five types of metabolic pathways were found to be significantly enriched: starch and sucrose metabolism, plant–pathogen interaction, MAPK signaling, plant hormone signal transduction, and biosynthesis of amino acids. Similarly, the purple module showed significant enrichment in the following five types of metabolic pathways: plant–pathogen interaction, MAPK signaling, biosynthesis of amino acids, ribosome, and carbon metabolism.

**Figure 8:**
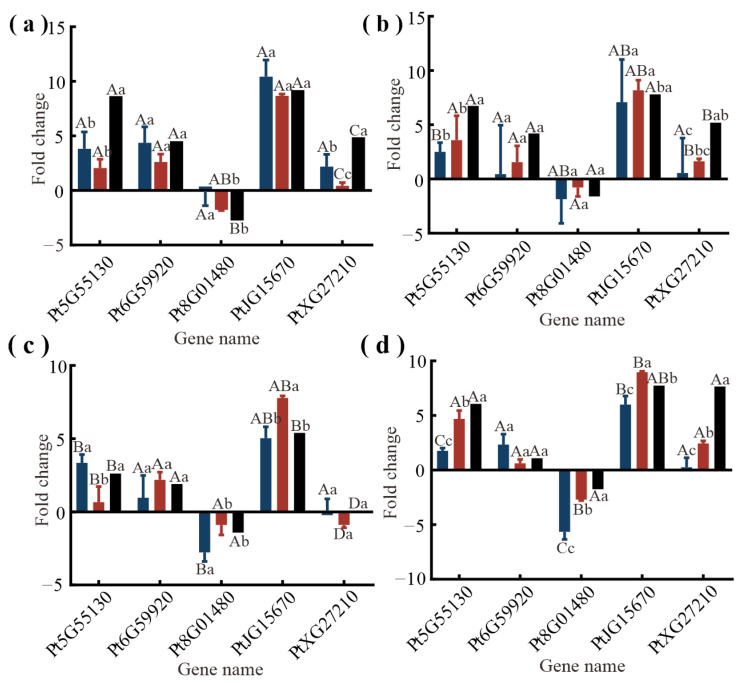
Gene expression levels from qPCR and RNA-seq. Blue columns represent the qPCR results (18s), red columns represent the qPCR results (actin), and black columns represent the RNA-seq results. Indicated by the y-axis is the fold change in the relative expression level of the gene between 0 h vs. FS 2 h (**a**), 0 h vs. FS 8 h (**b**), 0 h vs. LCC 2 h (**c**), and 0 h vs. LCC 8 h (**d**). Different lowercase letters indicate significant differences between the same gene using different internal references and transcripts at the same time (*p* < 0.05); different uppercase letters indicate significant differences between the same gene using different internal references and transcripts at different time (*p* < 0.05). The error bars indicate the means ± SD.

To verify the validity of the transcriptome results, five genes were randomly selected for q-PCR using either 18sRNA or actin as the reference. The results for the expression of these genes are consistent with RNA-seq. Moreover, the correlation coefficients were R^2^_18sRNA vs. RNA-seq_ = 0.77, R^2^_actin vs. RNA-seq_ = 0.80 (*p* < 0.01). These results indicate the reliability of RNA-seq (Figure 8).

In summary, gene co-expression modules are used for analyzing the functions and metabolic pathways of genes involved in feeding behavior. Additionally, a larger number of genes that are associated with signal transduction within the cell membrane and endoplasmic reticulum participate in MAPK and plant hormone signaling pathways. It is noteworthy that most of these genes are induced by mechanical damage, specifically inducing the activation of genes related to ribosomes and carbon metabolism. However, it is important to note that only specific inducers have the ability to trigger gene expression in the lumen of both the membrane and vacuole.

### 2.4. Differential Co-Expression of Genes and Metabolites Induced by Feeding Stimulation

**Figure 9:**
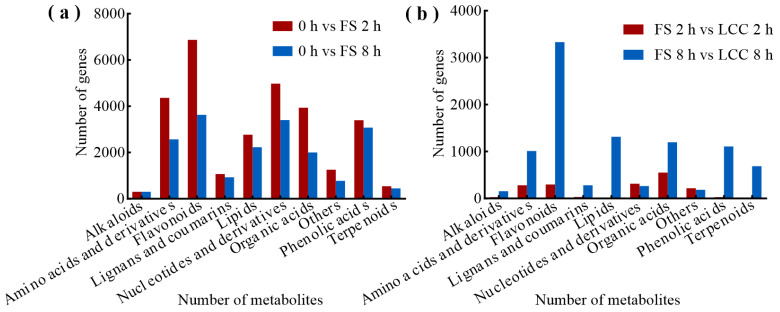
Number of genes significantly correlated with metabolites and the category in feeding stimulation comparison groups (**a**) and leaf clipping comparison groups (**b**).

To systematically study the interactions between the transcriptome and metabolome, calculations were performed regarding the relationships between all DEGs and DAMs (Figure 9). The results show that, in the 0 h vs. FS 2 h group, the highest number of genes are found to be significantly correlated with flavonoids, totaling 6873 genes. Next were nucleic acids and derivatives, as well as amino acids and derivatives. The number of genes correlated with the same class of metabolites was lower than in the 0 h vs. FS 8 h group than in the 0 h vs. FS 2 h group. Flavonoids remained the most closely related substances to the genes, with 3633 genes, which is lower than for the FS 2 h comparison group. In the mechanical damage comparison group, the gene–metabolite relationship was stronger in the FS 8 h vs. LCC 8 h group compared to the FS 2 h vs. LCC 2 h group. No genes related to terpenes and lipids were identified in the FS 2 h vs. LCC 2 h group.

### 2.5. JA Activates Flavonoid Expression

As flavonoid biosynthesis and plant hormone signal transduction were enriched at both transcription and metabolism levels, the differentially expressed protein (DEPs, based on proteomics of the same material) were related to pathways in order to clarify how these two pathways regulated resistant substances.

**Figure 10:**
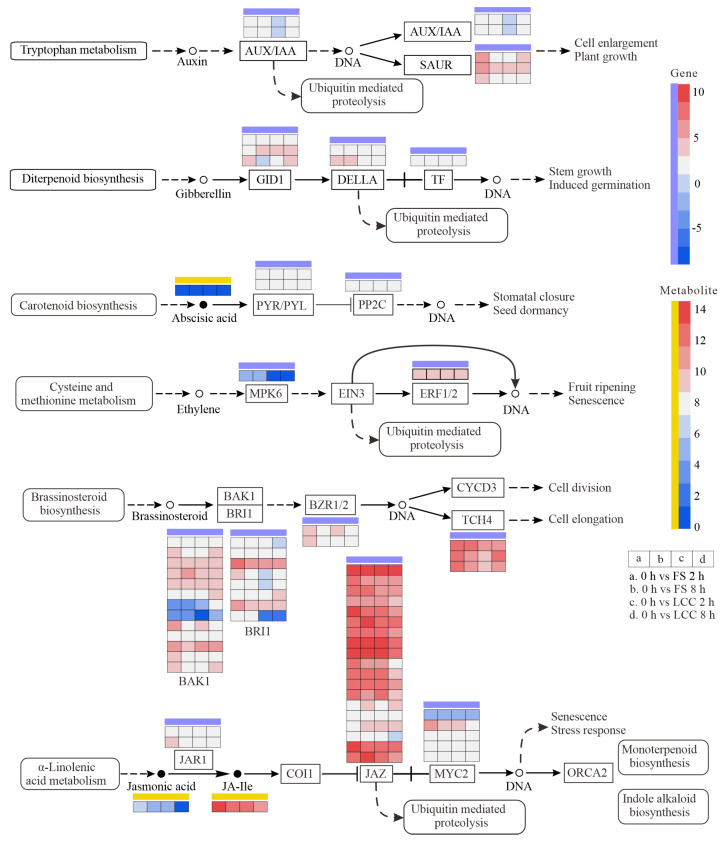
Changes in key genes and metabolites of plant signal transduction based on KEGG pathways. Expression is presented as log_2_FC heatmaps. Yellow bars indicate metabolites and blue bars indicate genes. The four colored squares are 0 h vs. FS 2 h, 0 h vs. FS 8 h, 0 h vs. LCC 2 h, and 0 h vs. LCC 8 h in turn.

The results show that proteins involved in plant hormone transduction are not significantly expressed, nor are the genes and metabolites in the SA pathway (Figure 10). This indicated an inconsistency in transcription and translation. In contrast to MPK6, JA and JA-Ile significantly differentially accumulated in FS and LCC groups, and most phytohormones were not found to be differentially expressed. Furthermore, metabolites participating in gibberellin (GA) and ABA signal transduction were upregulated in the FS comparison groups. Though a more significant difference was observed in JAZ after feeding stimulation, leaf clipping could also cause this trend. Expressions of AUX/IAA (*Pt0G31750* and *PtXG26430*) and BZR1/2 (*Pt3G39580* and *Pt3G39610*) were higher in 0 h vs. FS 2 h than 0 h vs. LCC 2 h, though this difference was not observed 8 h after the different treatments. Thus, these substances may all be serving as early responders to biotic stress.

**Figure 11:**
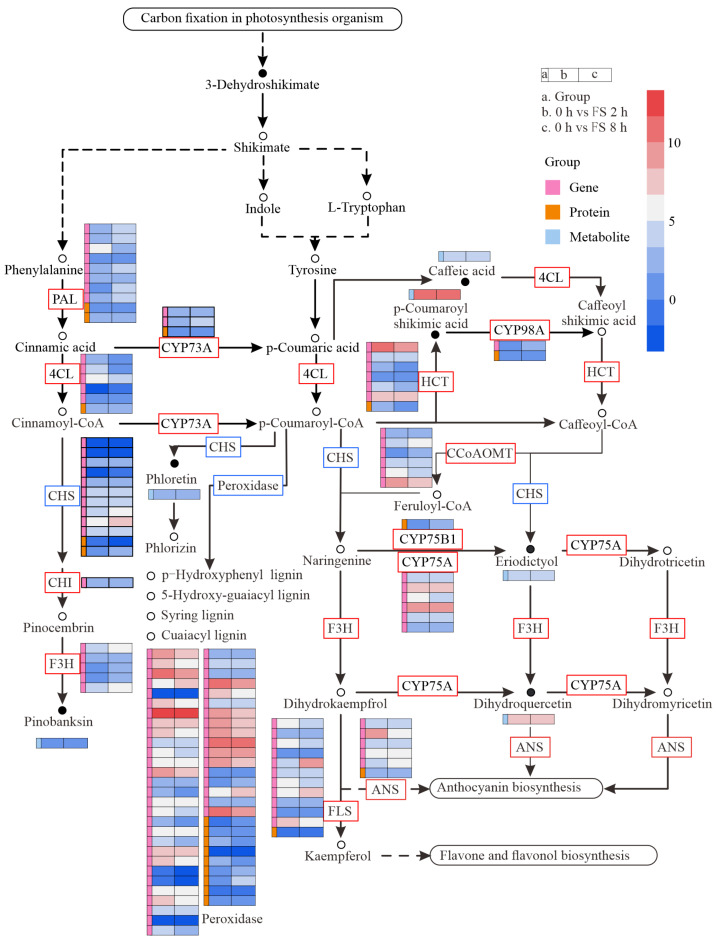
Changes in phenylpropanoids and flavonoids based on KEGG pathways. Expression is presented as log_2_FC heatmaps. Pink bars indicate gene transcripts. Orange bars indicate proteins. Blue bars indicate metabolites. The colored squares are groups of 0 h vs. FS 2 h and 0 h vs. FS 8 h.

In addition, phenylpropanoid and flavonoid biosyntheses were enriched in the two FS groups. In this process, shikimate is transferred to indole, then tyrosine and phenylalanine function together in generating phenylpropanoids and, finally, flavonoids. Phenylalanine ammonia-lyase (PAL, *PtJG02420.1* and *PtJG17740.1*), 4-coumarate-CoA ligase (4CL, *Pt8G27100.1*), cytochrome P450 family (CYP73A and CYP98A, *Pt5G34800.1*, and *Pt8G33570.1*), cinnamoyl-CoA reductase (CCR, *Pt8G52410.1*), hydroxycinnamoyl-CoA: shikimate hydroxycinnamoyl transferase (HCT, *PtQG28720.1*), caffeoyl-CoA-O-methyltransferase (CCoAOMT, *Pt7G02970.1*, *PtJG48410.1*, *PtJG48420.1*, and *PtJG48450.4*), chalcone synthase (CHS, *Pt3G29450.1*), and anthocyanidin synthase (ANS, *Pt5G55130.3*) were found to be upregulated in the results for both the transcriptome and proteome (Figure 11). Flavanone 3-hydroxylase (F3H) and chitinase (CHI), however, were only found to be enriched in the transcriptome results.

**Figure 12:**
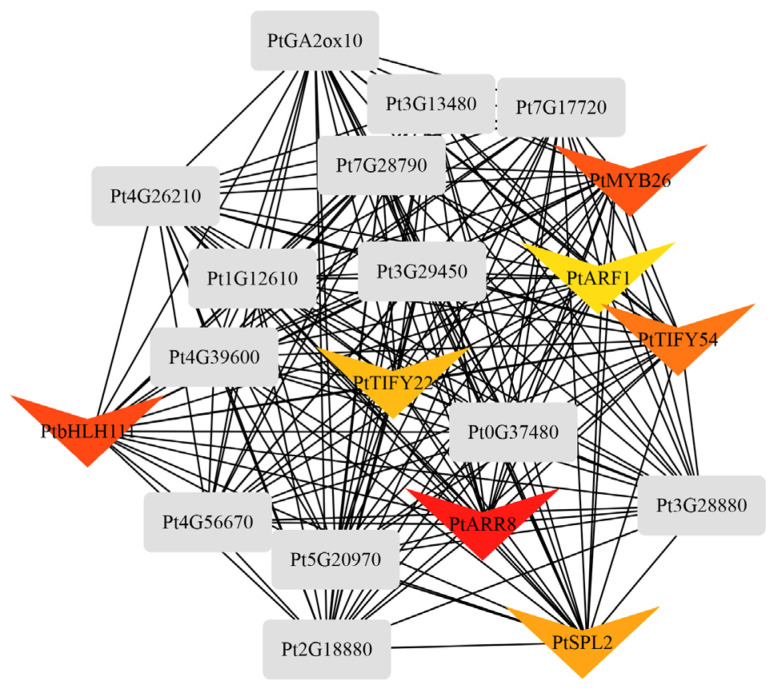
Interactions between genes involved in plant hormone signal transduction and flavonoid biosynthesis. Shape arrow present transcript factor. The darker the color means the strong interaction.

Due to changes in the plant hormone signal transduction pathway, the pathways related to flavonoid biosynthesis, starch and sucrose metabolism, and pentose and glucuronate interconversion are enriched in the black module. Similarly, the metabolic profile also indicates enrichment related to the plant hormone signal transduction pathway and flavonoid biosynthesis. Therefore, an interaction analysis was conducted on genes involved in these two pathways (Figure 12). The results show a close relationship between the plant hormone JA and flavonoid metabolism. Transcription factors *PtMYB26*, *PtTIFY54*, and *PtTIFY22* were found to participate in the interaction between these two pathways.

**Figure 13:**
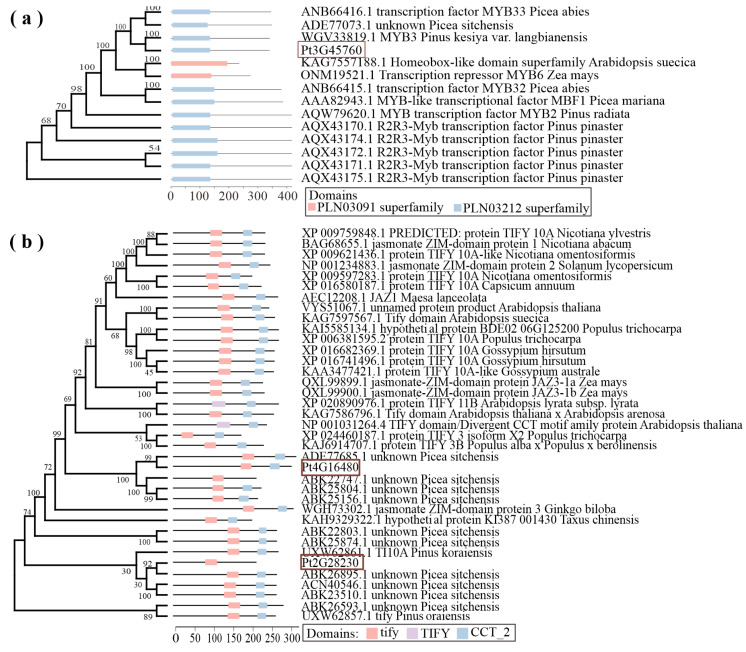
Phylogenetic tree and domain prediction of MYB (**a**) and TIFY (**b**) transcription factors. The transcription factors identified in this study have been marked with red frame: *PtTIFY22* (*Pt4G16480*), *PtTIFY54* (*Pt2G28230*) and *PtMYB26* (*Pt3G45760*).

Multiple sequence alignment analysis revealed that both the *PtTIFY22* (*Pt4G16480*) and *PtTIFY54* (*Pt2G28230*) genes in the black module contain TIFY and Jas domains. The results of phylogenetic trees constructed based on genetic differences with model plants and other pine species show that these two genes are homologous to TIFY genes in other pine species. Further analysis of their conserved domains reveals that the TIFY domain is found annotated in both *PtTIFY22* and *PtTIFY54*. Therefore, it is speculated that these two genes are evolutionarily conserved in Chinese pine and are involved in the formation of resistance against the Chinese pine caterpillar. The genetic phylogenetic tree and conserved domain annotation of *PtMYB26* (*Pt3G45760*) also suggest its involvement in resistance formation (Figure 13).

**Figure 14:**
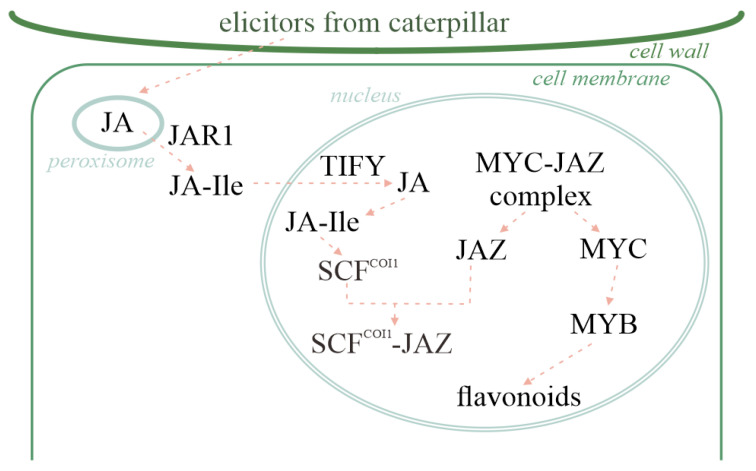
Plant hormone signal transduction and activation of flavonoid biosynthesis. Note: This figure shows only the interactions of these substances and does not indicate a direct reaction.

In the feeding stimulation groups, we found significantly higher differences between JA, JAZ, and JA-Ile than between other plant hormones. The results of both transcriptome and metabolome analyses, as well as the combined analysis, show that there is a significant enrichment of DEGs and DAMs in the flavonoid synthesis pathway. Moreover, there is also an interaction between these two metabolic pathways. Therefore, based on the expression levels and cellular localization, we can preliminarily infer the signal transduction pathway shown in Figure 14. After pine caterpillar feeding, JA in the peroxisomes of pine leaf cells first increases and, due to the catalysis of JAR1 in the cytosol, JA-Ile is then generated and enters the nucleus. Due to JA feedback regulation, a small amount of JA-Ile induces the expression of TIFY transcription factors in the nucleus, activating JA generation in the nucleus. A large amount of JA-Ile is generated in the nucleus, promoting the formation of a complex between SCF^COI1^ and JAZ, resulting in the release of MYC from the transcription factor-binding region. The predicted mutual interaction shows that there is an interaction between the MYC and MYB transcription factors, suggesting that they may be key substances for the generation of phenolic compounds in the plant hormone signaling pathway for the flavonoid pathway.

## 3. Discussion

### 3.1. Different Responses Induced by Feeding Stimulation and Leaf Clipping Control

How do plants recognize mechanical damage and herbivory, and how does this determine the defense mechanisms they subsequently activate? This is reflected by differences in the expression of certain genes and the various substances that accumulate. In this study, we conducted gray relational analysis of plant hormones and performed metabolomics profiling. The results show that herbivory by the pine caterpillar induces significant changes in the expression of substances within the plant, and these differences are also significant when comparing them with simple mechanical damage. Therefore, herbivory by the pine caterpillar is the primary cause of the changes in substances in the pine tree.

Exploring the differences between feeding and mechanical damage contributes to understanding the induction of plant-specific resistance, thereby enhancing our knowledge of plant defense mechanisms. In tomato plants subjected to feeding by beet armyworms and mechanical damage, the expression of PPO was significantly higher after feeding, a difference in induction due solely to elicitors present in the oral secretions of beet armyworms [24]. Cotton plants treated with oral secretions from beet armyworms showed upregulations of monoterpenes and sesquiterpenes, whereas no such differential expression was observed in mechanically damaged plants [25]. Chewing insects feeding on foliage and pathogen infections can trigger an oxidative burst, leading to *Vm* and sustained reduction for a period during which H_2_O_2_ is released [14]. Experiments indicate an increase in reactive oxygen species levels soon after feeding, and we observed a significant upregulation of catalase gene expression in both comparison groups after feeding, confirming a substantial enzymatic response to mitigate damage from reactive oxygen species induced during feeding. Subsequently, the expression of resistance substances acting on insects is induced.

Because mechanical damage alone is insufficient to induce a complete response, and the elicitors present in oral secretions can enhance this response, the application of oral secretions (OSs) to tobacco plants grown in the field triggers an outbreak of JA-Ile [26]. Thus, during the induction of plant resistance, certain substances are expressed in the early stage to act on oxidative stress, leading to the formation of resistance. This study explored the mechanism of induced resistance in plants under natural conditions following insect threat. The results reveal significant differences in the plant transcriptome and metabolome due to feeding treatment and the simulation of mechanical damage by leaf clipping. Therefore, research on plant–insect interactions need not be confined to indoor experiments, but can be conducted directly under the natural growth conditions of plants. This approach not only preserves the authentic growth environment, but also allows for the control of experiment variables. However, this study relied solely on leaf cutting to stimulate damage, and despite numerous experiments utilizing oral secretions extracted from insects [27], there is a lack of control experiments involving oral secretions for used in confirming the specific components and forms crucial for plants in distinguishing between mechanical damage and insect feeding. Moreover, further experimental evidence is needed to ascertain whether the plant receptors identified to date are evolutionarily conserved in conifers and thus capable of recognizing insect elicitors.

### 3.2. The Role of Plant Hormones in Signal Transduction Related to the Induction of Plant Resistance to Insects

Insects’ feeding behavior leads to significant differences in plant signaling. Previous studies have shown that JA signaling plays a crucial role in the interaction between plants and insects during feeding. However, it is evident that plants require more than one hormone for coordinating growth and resistance.

During periods of short-term stress, plants produce a large amount of JA. JA-Ile can bind to the COI1 receptor, mediating the ubiquitination and degradation of JAZs, thereby activating the JA signaling pathway and regulating most steps of anthocyanin formation in the flavonoid pathway [28,29]. A high expression of JA was observed between 0.5 h and 2 h after treating different varieties of tobacco with HAE. Changes in the metabolome corresponding to the flavonoid pathway were observed at 2 h, indicating that feeding by pine caterpillars may induce JA expression and regulate the subsequent involvement of the flavonoid pathway in oxidative stress. Differences in the expressions of JA and JA-Ile were observed between 2 h and 8 h following treatment, being consistently higher in FS treatment than LCC treatment. This is in accordance with the understanding of plant hormone function, namely, the JA content rapidly increases during short-term feeding stimulation and then recovers to levels not significantly different from untreated plants, thus avoiding an excessive accumulation of JA, which is potentially detrimental to plant defense and growth development.

JA plays a crucial upstream role in the synthesis of terpenoids, which are involved in various aspects of plant resistance to insects, including toxicity to insects, attraction of predatory insects, and induction of early defense in neighboring plants [30]. Plants exposed to volatile organic compounds (VOCs) produce higher levels of aromatic and terpenoid volatiles within 180 to 300 min after defense induction. The initiation of VOC-induced defenses is controlled by JA [31]. The regulation in JA expression was more pronounced following feeding stimulation than in the leaf clipping control. Terpenoids are important substances for the resistance of conifer trees to insects; not only do they serve as aromatic compounds representing the developmental period of plants [32], but experimental evidence has shown that plant secondary metabolites, such as terpenes, infiltrate into the soil through leaf litter, altering the activity of enzymes in the soil and participating in soil decomposition. Currently, knowledge regarding the metabolism of terpenoid substances is rather comprehensive [33,34]. From the KEGG enrichment analysis, an enrichment in DAMs involved in terpenoid biosynthesis was found. The high content of terpenoid compounds is also one of the characteristics that distinguish resistant *Picea* from the control group [35]. In Chinese pine, the compounds β-pinene, trans-caryophyllene, and limonene have been identified as participating in resistance against red turpentine beetles [36].

Glucosinolates are important secondary metabolites in Brassicaceae that serve as a defense mechanism against herbivorous insects. It has been shown in Arabidopsis that fluctuations in JA/JA-Ile levels induced by insect feeding can result in glucosinolates being transported from mature to young leaves [37]. However, in this study, it was observed that glucosinolate expression was upregulated upon leaf clipping, but not significantly enriched following feeding stimulation. Mao also suggested that the increase in glucosinolate levels induced by insect feeding is more significant in older Lepidoptera plants [38]. In our study, the age of the plants used in the treatment and control groups was consistent, yet there were still differences in hormone levels between the LCC and FS groups. The reason for this difference may be the different methods of treatment, but research is still lacking regarding on the changes in glucosinolate levels induced by mechanical damage that trigger the plant hormone JA.

The differences in the expression of substances in the SA pathway mostly occur in plants after pathogen attacks, but insects can also induce synergistic SA and JA signaling transduction; plants can also preemptively initiate resistance to pathogens through the SA pathway [39]. SA can be generated in plants through two pathways involving the synthesis of either phenylalanine or isovaleric acid, both of which use p-coumaric acid as a reaction precursor. In this study, there was no significant difference in SA and its analog 4-hydroxybenzoic acid, which were identified from the result of hormone determination and metabolomics. This is consistent with a previous conclusion that there are significant changes in SA expression in plants under piercing–sucking insect stress. For example, changes in genes for WRKY40 and WRKY70 homologs involved in SA signal transduction were identified in pepper plants after feeding by the two-spotted spider mite [40]. The latest experiments on *Arabidopsis thaliana* indicate that SA generation is not fully completed through the phenylpropanoid pathway [41]. This discrepancy may be attributed to the upregulation of related genes in the phenylpropanoid pathway during this study, despite the lack of significant differences in SA content. However, further research is needed to confirm whether this is related to species differences in conifers. SA has the ability to interfere with the transportation of certain proteins, indicating an immediate response timeframe of 10 s to 10 min, which is much shorter than the time that is possible for transcriptional regulation. As a result, Yan and Dong suggest the presence of receptors within cells that can mediate the early response of SA [42]. Consequently, during the initial stages of HAMPs, SA can function as a messenger compound. However, the identification of early response receptors necessitates more precise timing and accurate processing to differentiate the early responses induced by HAMPs from the damage caused by mechanical injury to plants.

### 3.3. Flavonoid Biosynthesis Involved in Plant-Induced Resistance to Insects

Some plant hormones have been found to be involved in regulating the synthesis of flavonoids [43]. In non-stress conditions, flavonoids play a role in the development of plant organs and seeds, such as the germination and growth of pollen tubes or the maturation, dormancy, and longevity of seeds.

Flavonoids participate in plant response to stress by scavenging free radicals and exerting antioxidant effects [44,45,46,47]. In this study, a significant accumulation of DAMs in the flavonoid pathway was observed in both treatment comparison groups. According to the KEGG metabolic pathway, C4H in the phenylalanine pathway hydrolyzes cinnamic acid into 4-coumaric acid [48], and p-coumaroyl-CoA and naringenin chalcone are then generated under the catalysis of chalcone synthase [49]. These compounds serve as precursors for the synthesis of most of the flavonoids found to be differentially expressed in this study, but no significant difference in expression levels was observed after 8 h of different treatments. After feeding stimulation for 8 h, levels of the downstream product naringenin chalcone were significantly higher than in the mechanical damage treatment, while expression levels of the downstream metabolite naringenin chalcone increased 2 h after leaf clipping. The extended feeding time of the pine caterpillar weakened the flow of limonene to naringenin, which under the catalysis of flavanone 3-hydroxylase (F3H) resulted in the formation of dihydroflavonols, such as dihydroquercetin, dihydromyricetin, and dihydrokaempferol [49]. In alfalfa, graphene can induce the upregulated expression of dihydroquercetin and proline to participate in ROS clearance in the plant and protect it from oxidative damage [50]. These results are consistent with the changes in metabolites in sugarcane after *Mythimna separate* (Walker) feeding and in tea trees following caterpillar feeding [51,52]. Niu et al. believed that the increase in anthocyanin content in plant tissues could change the color of plants, allowing them to avoid becoming the target of insects [53]. In the experimental process of this study, a difference in color was observed between the feeding points of the pine caterpillars on the pine needles and the color of cut leaves, indicating that the pine trees had an increase in anthocyanin content after feeding, which is consistent with the results of the metabolome analysis. However, further experimental research is required to determine whether the high expression of anthocyanins observed in this study reaches the levels required to deter pine caterpillars.

Caterpillar feeding induced large amounts of flavonoids and phenylpropanoids in pine trees, in addition to various modifications that participate in plant oxidative stress. Plants perceive stress and produce varying degrees of oxidative stress [54], with upregulation in the activity of enzymes, such as LOX, SOD, and CAT, which promote JA synthesis. In response to insect infestation, the generation of large amounts of reactive oxygen or upregulated expression of antioxidant enzymes is observed in conifers such as pine [55] and spruce [36]. Differential expressions of antioxidant enzyme genes and proteins are also observed in pines (Figure 11). TIFYs represent are a class of widely distributed plant transcription factors that operate in the cell nucleus in regulating the gene expression of key enzymes in the JA biosynthesis pathway [56]. JAZ1/TIFY10A is one of the JAZ repressor proteins involved in JA signal transduction [57,58]. One possible mechanism by which jasmonate (JA) interacts with flavonoids is to induce the expression of MYB transcription factors, while flavonoids can bind to and regulate the activity of MYB. The interaction between JA and MYB transcription factors is mediated by the COI1 receptor. COI1 is a key factor in the JA signaling pathway that binds to JA to form a COI1–JA complex, promoting the degradation of JAZ proteins through the ubiquitin/26S proteasome pathway [59]. This leads to the release of the transcription factor MYC2, which regulates the expression of jasmonate-responsive genes [60]. JAZ proteins negatively regulate MYB transcription factors, as their degradation promotes the activation of MYB transcription factors [61]. Through this mechanism, JA can regulate the activity and expression of MYB transcription factors, thereby affecting plant growth and development [29]. The expression of key enzyme genes in the flavonoid pathway, such as flavonol synthase (FLS), dihydroflavonol reductase (DFR), anthocyanidin synthase (ANS), and UDP-glycose flavonoid glycosyltransferase (UFGT), is regulated by MYB transcription factors [62,63]. In different plants, MYB transcription factors have different regulatory mechanisms for the genes of key enzymes of flavonoid metabolism. For example, in alfalfa, MsMYB741 is an important regulatory factor in flavonoid biosynthesis, directly regulating the expression of phenylalanine ammonialyase 1 (MsPAL1) and chalcone isomerase (MsCHI) genes [64]. In tea trees, CsMYB2 and CsMYB26 regulate anthocyanin synthesis by regulating the expression of CsF3’H and CsLAR [65]. MYB transcription factors can also bind to the promoter regions of CYP genes to stimulate or inhibit their transcription and expression [66,67]. Previous studies have shown that the CYP75A gene, which encodes a member of the cytochrome P450 protein family, can confer resistance to cotton bollworm in plants [68,69]. In this study, differential expressions of PtCYP73A, PtCYP98A, PtCYP75A, and PtCYP75B1 were observed (Figure 11). The CYP family also plays an important role in the process of plant response to biotic stress through participation in receptor-like kinases (RLKs) and mitogen-activated protein kinase (MPK) signaling pathways. This is also supported by the significant enrichment of participants in the MAPK signaling pathway in the black module (Figure 7). The activation of MPK2 is dependent on MKK3 and COI1, further demonstrating the important role of JA in the damage-triggered activation of MPK2 [70]. There are multiple ways for plants to remove ROS [60]. The plant hormone ABA interacts with G proteins to respond to oxidative stress [71,72]. As shown in Figure 10, the expression of ABA is upregulated. Studies have shown that the MYC transcription factor in the JA pathway is co-regulated by ABA in resistance to chewing insects [73]. However, there was no significant difference between the leaf clipping control and feeding stimulation, indicating that mechanical damage caused by chewing insects during the feeding process is an important factor in ABA variation. Furthermore, there is an upregulated expression of the upstream genes JAZ, DELLA, and AUX/IAA involved in ubiquitination, suggesting ubiquitination and glycosylation are required in plant hormone-mediated signal transduction. Additionally, glycosylation is necessary for the antioxidant activity of flavonoids [72,74]. The identity of the 47 genes and 9 proteins found to be differentially expressed in this study further validates the occurrence of oxidative stress response.

In addition to participating in oxidative stress, flavonoids play a crucial role in the formation of plant resistance in various other ways. Lignin, which is the primary component of plant cell walls, poses difficulties for insects in terms of chewing and digestion [75,76]. Previous studies have demonstrated the involvement of lignin in insect resistance in rice [77], chrysanthemum [78,79], and cassava [80]. Lignin expression is observed to be significantly higher in the resistant stone cells of *Picea*, providing protection against the elephant beetle [81]. Figure 11 shows that genes associated with lignin synthesis, such as *PtPAL*, *Pt4CL*, *PtHCT*, *PtCCoMOAT*, and *PtCCR1*, are upregulated at the transcriptional level upon insect feeding, contributing to the formation of resistance. The mechanical damage caused by chewing activates plant hormone signaling transduction [82], while saliva can also increase the lignin content in cells, thereby enhancing resistance [83,84,85]. In cotton, there is a concurrent increase in JA and decrease in lignin content [86]. In this study, a high expression of lignin was not detected under conditions where there was high expression of the related synthetic enzymes. Therefore, besides the mechanical damage caused by chewing insects during the feeding process, specific saliva inducers or certain behaviors, even the order or accumulation, should be considered when exploring reasons for the high expression of substances related to lignin synthesis. Additionally, tannins [87,88] enter the insect’s intestinal tract during insect feeding, forming quinone substances that lead to insect sterility or death.

Insects feeding can trigger defensive reactions in plant, such as in the case of PAL overexpression in *Ricinus communis* leading to dwarfism [89]. Oak trees also exhibit reduced photosynthesis under the feeding of *Lymantria dispar* [90], but phenolic substances do not affect the growth of white spruce [91]. In other plants, insect feeding may actually enhance photosynthesis [92]. Due to cross-talk between plant hormones, IAA, ET, brassinosteroids (BRs), and GA pathways were upregulated in this study, but further investigation is still required to determine their specific modes of action.

## 4. Materials and Methods

### 4.1. Study Area and Experiment Setting

The study area is located in Huangtuliangzi Forestry, Pingquan City, Chengde City, Hebei Province, China (41°18′ N, 119°13′ E), where there is a mid-temperate continental dry monsoon mountain climate, and the annual rainfall is about 540 mm. Healthy Chinese pines in pure forests with the same altitude (560 m), slope direction and slope, similar tree vigor and management, and with no pine caterpillar occurrence were selected for treatment. Treatments were carried out using 10 caterpillars and employing a leaf clipping control to simulate mechanical damage.

Before the initiation of experiments, pine caterpillars aged five to seven instar were starved for 10 h. The insects were collected at Qigou Forestry, Pingquan City, Chengde City, Hebei Province, China (40°55′ N, 118°21′ E). The selected pines were about 10 years old and were divided into three groups for the following treatments: feeding stimulation, leaf clipping control, and CK. Under the natural condition, there were two feeding stimulation (FS) groups, where 10 caterpillars were placed on the plants, and for the two leaf clipping control (LCC) groups, needle tips were repeatedly cut off to simulate foraging by 10 caterpillars, and there was one CK group. Each of the groups was also divided into 2 and 8 h after treatment to form the FS 2 h, FS 8 h, LCC 2 h, and LCC 8 h groups. Timing began with the placement of caterpillars in the FS group and with the first cutting of needles in the LCC group. For CK, treatment was 0 h. The operations were on the branch with consistent length and ground clearance in the north, south, east, and west, and needles were taken 3 cm below the chewing sites or the cutting points. Ten needles per branch were collected as a single sample using tin foil, then immediately plunged into liquid nitrogen, followed by transfer to a −80 °C freezer for storage.

### 4.2. Quantifications of JA and SA

Pine needles (0.6 g) were ground in 5.4 mL of phosphate buffered saline (PBS) (pH = 7.3) using liquid nitrogen. Following centrifugation at 5000 r/min for 30 min at 4 °C, the supernatant was collected. JA and SA contents were detected using Plant Hormone ELISA Kits (Shanghai Enzyme-linked Biotechnology Co., Ltd., Shanghai, China). The kit employs a double-antibody sandwich method, and the optical density values were measured at 450 nm using a microplate reader. All *r*^2^ values of the standard curves were higher than 0.9, indicating a good correlation with the model. The correlation degree was calculated using gray relation analysis (GRA). Gray relation analysis was used to evaluate which factor had the greatest impact on a target indicator. It has been used in some macrostudies, such as in environmental science [93] and healthcare [94]. Compared with the traditional methods, where only the relationship between independent and dependent variables is calculated, GRA can capture the nonlinear relationships between data.

### 4.3. RNA Preparation

Total RNA was extracted from pine needles using the TRIzol kit after grinding, sedimenting, and cleaning of samples. RNA degradation and contamination were then monitored on 1% agarose gels. RNA purity was checked using the NanoPhotometer^®^ spectrophotometer (IMPLEN, Westlake Village, CA, USA). RNA concentration was measured using the Qubit^®^ RNA Assay Kit and the Qubit^®^2.0 Fluorometer (Life Technologies, Carlsbad, CA, USA). RNA integrity was assessed using the RNA Nano 6000 Assay Kit of the Bioanalyzer 2100 system (Agilent Technologies, Santa Clara CA, USA). The total RNA was used for transcriptome assay and q-PCR analysis.

### 4.4. Transcriptome Assay

The total RNA of pine needles of the five groups (0 h, FS 2 h, FS 8 h, LCC 2 h, and LCC 8 h) was used for library preparation. Sequencing libraries were generated using the NEBNext^®^Ultra^TM^ RNA Library Prep Kit for Illumina^®^ (San Diego, CA, USA) following the manufacturer’s recommendations and index codes were added to attribute sequences to each sample. The original data were filtered using fastp v 0.19.3. All subsequent analyses are based on clean reads. The reference genome and its annotation files were download (NCBI accession: PRJNA784915), with HISAT v2.1.0 used to construct the index, and clean reads were compared to the reference genome. Feature Counts v1.6.2 was used for gene alignment and to calculate FPKM. DESeq2 v1.22.1 was used to assess the differential expression between the two groups, and the *p*-value was corrected using the Benjamini and Hochberg method. The corrected *p*-value and log_2_|Fold Change| were used in thresholding to identify the differentially expressed genes (DEGs).

### 4.5. Metabolome Assay

The identified peaks were qualitatively identified based on the self-built database MWDB (Metware database) and the metabolite public database. Finally, the isotope signals, repetitive signals containing K^+^, Na^+^, and NH_4_^+^, and other repetitive signals were removed. Primary and secondary metabolites of pine needles with different treatment modes were analyzed using an UPLC-ESI-MS/MS system (UPLC, SHIMADZU Nexera X2 https://www.shimadzu.com.cn/, accessed on 7 June 2024; MS, Applied Biosystems 4500 Q TRAP). The data were unit variance scaled prior to subsequent bioinformation analysis. Differentially accumulated metabolites (DAMs) were determined according to variable importance in projection (VIP) and log_2_|Fold Change|.

### 4.6. q-PCR Analysis

The SYBR Green reaction system was used for PCR reactions, with 18sRNA [95] and actin [96] used as internal references. The relative gene expression was calculated based on the 2^−∆∆CT^ method. Duncan’s new multiple range test was used for significance testing (*p* < 0.05). Information regarding the PCR system, reaction program settings, and primer sequences is shown in Supplementary Table S1.

### 4.7. Statistic Methods

fastp v 0.19.3 was used to filter the original transcriptome data. PCA was performed using the statistics function prcomp within R (www.r-project.org, accessed on 7 June 2024). DESeq2 v1.22.1/edgeR v3.24.3 was used to analyze the differential expression between the two groups, and the *p*-value was corrected using the Benjamini and Hochberg method. The enrichment analysis was performed based on the hypergeometric test. For KEGG, the hypergeometric distribution test was performed using the unit of pathway; for GO, it was performed based on GO terms. WGCNA v1.69 was used for weighted gene co-expression network analysis. The determination of co-relationships between q-PCR and transcripts was conducted by DPS v7.05, and the *p*-value was calculated using Duncan’s new multiple range test.

## 5. Conclusions

Through the analysis of differential expression in the transcriptome and metabolome of needles of *Pinus tabuliformis* under natural conditions, including after being fed by pine caterpillars, it was found that pine can precisely distinguish between the mechanical damage caused by insect feeding and mechanical damage, even in a complex natural environment, and respond with apparent regularity through plant hormone expression, signal transduction, synthesis of specific resistance substance synthesis, and differential gene expression. In the process of pine forming resistance in response to chewing, JA induces the biosynthesis of flavonoids and terpenoids as signaling compounds, while TIFY transcription factors participate in specific metabolic pathways of needle trees that are part of a dedicated response to feeding by mandibulate insects.

## Figures and Tables

**Table 1:** Correlation degree and rank of treatment mode and time after treatment.

Plant Hormone	Evaluation Items	Correlation Degree	Rank
JA	Treatment mode	0.745	1
	Time after treatments	0.595	2
SA	Treatment mode	0.822	1
	Time after treatments	0.5	2

## Data Availability

The transcriptome data have been submitted to NCBI (PRJNA1006455).

## Supplementary Materials

The following supporting information can be downloaded at: https://www.mdpi.com/article/10.3390/ijms25126354/s1.
